# Editorial: Extracellular vesicles in cancer immunosurveillance

**DOI:** 10.3389/fimmu.2022.993967

**Published:** 2022-08-04

**Authors:** Milad Moloudizargari, Mohammad Hossein Asghari, Malene Møller Jørgensen, Russel J. Reiter, Dieter Kabelitz

**Affiliations:** ^1^ Beckman Research Institute of City of Hope, Duarte, CA, United States; ^2^ Department of Pharmacology and Toxicology, School of Medicine, Babol University of Medical Sciences, Babol, Iran; ^3^ Department Clinical Immunology, Aalborg University Hospital, Aalborg, Denmark; ^4^ Department Clinical Medicine, Aalborg University, Aalborg, Denmark; ^5^ Department of Cell Systems and Anatomy, UT Health San Antonio, Long School of Medicine, San Antonio, TX, United States; ^6^ Institute of Immunology, University of Kiel, Kiel, Germany

**Keywords:** exosome, tumor, cancer, immunosurveillance, tumor microenvironment

Accumulating evidence suggests that a considerable proportion of cancer-host interactions are mediated through extracellular vesicles (EVs), small membranous structures produced by a wide variety of cell types. Being initially praised for their immunological inertness and stealth, later years of research uncovered more aspects of their immunomodulatory properties leaving a great deal of ambiguities unaddressed.

From a cancer immunology point of view, EVs can be simply looked upon in two major categories: 1) EVs released by cancer cells, the so called tumor-derived EVs (TDEVs) and, 2) EVs produced by immune subsets and other related cells. TDEVs are of particular interest since they play critical roles in cancer progression *via* promoting cancer-intrinsic mechanisms of progression as well as through training the microenvironment and/or skewing the immune response toward a less effective anti-cancer entity. The latter has been especially highlighted by the ability of TDEVs to suppress the cytotoxic immune subsets not only *via* carrying regulatory molecules such as PD-L1 but also through their induction in other recipient cells in the tumor milieu (1–3). Another well-studied example of TDEV-mediated immunomodulation is the effects of EVs on natural killer (NK) cells, which have both suppressive and stimulatory effects based on the type of immune response in question as well as the type and duration of exposure. In this regard, the interactions of the activating receptors on the surface of NK cells, mainly NKG2D, with their ligands such as MIC-A, are affected by the EVs released by cancer cells (4). Herein, multiple myeloma in which NK cells play major protective roles, has been used as a suitable model to study the effects of cancer exosomes (5). We previously showed that myeloma exosomes, a better-described subset of EVs ranging between 30-150 nm in size (6), suppress the cytotoxic activity of NK cells partly through the downregulation of NKG2D expression on their surface, while they can also stimulate the production and release of IFN-γ from NK cells (4). In line with our previous findings, a recent study by Vulpis et al. revealed that despite the stimulatory effects of myeloma-derived exosomes on NK cells at early exposures, chronic exposure exerts suppressive effects (7), supporting the dual role of TDEVs as immunoregulators. Based on their findings, the NK-mediated shedding of NKG2D-activating ligands is not only employed by myeloma cells as a means of escape from NK-mediated immunity, but also may suppress NK function through the downmodulation of NKG2D on these cells and/or induction of NK cell fratricide (7).

Revealing further tumor-promoting aspects of TDEVs, Wang et al., in their interesting review of literature, discussed the role of TDEVs in mediating cancer lymphangiogenesis as a key process leading to lymph node metastasis (LNM). LNM is a major problem and prognosticator of poor outcome in many cancers for which currently there is no targeted drug in clinical practice. This raises the important questions that 1) “Can TDEVs be targets of future drugs against cancer LNM?” 2) “Can TDEVs be used as reliable sources of biomarkers to predict and monitor LNM?”. Despite the increasing number of efforts to elucidate such aspects of TDEVs, these questions still remain to be answered.

An ambitious approach toward exploiting the existing knowledge on the deteriorative roles of TDEVs is the therapeutic targeting of their biogenesis and function (8, 9). In line with this goal, Novais et al. discuss in their interesting review that melatonin as a molecule with overlapping functions with EVs can be a potentially attractive molecule for this purpose. Nonetheless, a major challenge related to the ambitious goal of therapeutically targeting TDEVs is the specificity of targeting these disease-promoting EVs without interfering with the normal physiological processes mediated by host EVs. Pursuing this aim, a deeper knowledge on the biogenesis of exosomes and the differences of their characteristics and cargo that result from certain pathology-driven milieu may aid in finding more specific and less toxic strategies to target the harmful EVs. The study of Jiang et al. is a good example of such efforts in which the authors comprehensively reviewed the effects of hypoxia, a well-studied condition associated with several pathologies, particularly cancer. They have discussed the changes in the EV-associated cargo as a result of cancer-related hypoxic condition and how it affects their immunomodulatory cargo.

In addition to TDEVs, the second major category of EVs are those produced by immune subsets. In this context, EVs released by cytotoxic T lymphocytes (CTLs) or NK cells are closely related to the intracellular vesicles involved in the cytotoxic effector functions. Herein, Lettau and Janssen discussed that due to the similarities of the lysosome-related EVs (LREVs) (involved in cytotoxic effector functions) with the EVs isolated from these cells, they may serve as the distant effector arms. In support of this notion, it has been revealed that in addition to the classical cytolytic effectors, LREVs also carry various immunomodulatory molecules such as MHC I and II as well as costimulatory and adhesion molecules, which can be also found on exosomes. These findings indicate that both intra- and extra-cellular vesicles are closely related and may function as distant effector processes of both innate and acquired immunity. This is particularly important since these findings could be potentially exploited to enhance the efficacy of immunotherapy through various strategies including but not limited to the use of engineered and untouched immune cell-derived EVs as cancer vaccines and boosters of the immune response (10).

Altogether, despite the growing knowledge on the biology and effector functions of EVs in cancer and their roles as modulators of the immune response, the translation of this knowledge into clinically feasible therapeutic and diagnostic strategies remains nascent, calling for further translational research in the field.

## Author contributions

A draft of the manuscript was prepared by MM. All authors contributed to the finalization of the manuscript and approved the submission.

## Conflict of interest

The authors declare that the research was conducted in the absence of any commercial or financial relationships that could be construed as a potential conflict of interest.

## Publisher’s note

All claims expressed in this article are solely those of the authors and do not necessarily represent those of their affiliated organizations, or those of the publisher, the editors and the reviewers. Any product that may be evaluated in this article, or claim that may be made by its manufacturer, is not guaranteed or endorsed by the publisher.
